# Outcome and survival analysis of pulmonary metastasectomy for primary sarcoma with pulmonary metastases

**DOI:** 10.3389/fsurg.2024.1470784

**Published:** 2024-10-30

**Authors:** Chih-Hsiang Chang, Xu-Heng Chiang, Mong-Wei Lin, Shuenn-Wen Kuo, Pei-Ming Huang, Hsao-Hsun Hsu, Jin-Shing Chen

**Affiliations:** ^1^ Department of Surgery, National Taiwan University Hospital, Taipei, Taiwan; ^2^ Department of Medical Education, National Taiwan University Hospital, Taipei, Taiwan

**Keywords:** sarcoma, pulmonary metastasis, lung metastasectomy, soft tissue, survival rate

## Abstract

**Background:**

Sarcomas are rare malignancies, accounting for approximately 1% of all cancers. Pulmonary metastases are the most preferential site for distant metastasis in malignant soft tissue sarcomas. Despite the lack of evidence from large randomized trials to support treatment guidelines, surgical resection of resectable metastatic tumors remains the current standard of care. This study aimed to explore the survival status of patients with soft tissue sarcoma after resection of pulmonary metastases.

**Methods:**

This study is a retrospective analysis of patients who mestastasectomy by means of lobar or sublobar resections at National Taiwan University Hospital and its branches. The statistical and investigation period was from February 2007 to December 2020.

**Results:**

Among 110 samples during the investigation period, the overall 5-year survival rate was 62.9%, which was higher than the 15%–50.9% reported previously. A disease-free interval of more than 12 months and the occurrence of local recurrence of sarcoma at the time of resection of pulmonary metastases are associated with overall survival. Most of the samples were treated with minimally invasive surgery (VATS), and therefore, most patients had a shorter hospital stay and better postoperative recovery.

**Conclusion:**

For pulmonary metastatic sarcoma, pulmonary metastasectomy is a relatively safe treatment method with short hospital stay and short ICU stay. The results of this study suggest that VATS is preferred over thoracotomy, but further observations are needed to confirm these findings.

## 1 Introduction

Sarcomas are a heterogeneous group of malignant tumors that include more than 70 different types, primarily arising from bone or soft tissue. The clinical manifestations of malignant soft tissue sarcomas in particular are often nonspecific, which often leads to delayed diagnosis. Therefore, by the time of detection, the tumor has usually progressed significantly and may have metastasized to distant organs (1). Soft tissue sarcomas (STS) are malignant soft tissue tumors that include multiple histological subtypes (e.g., liposarcoma, rhabdomyosarcoma, synovial sarcoma, leiomyosarcoma, and fibrosarcoma) that can occur almost anywhere in the body. STS mainly occurs in the lower limbs, followed by the upper limbs and trunk. Other common locations include the head, neck, and retroperitoneal space (2). Late-stage STS often metastasizes through the bloodstream, lymphatic system, and intra-abdominal pathways, which is an important reason for the poor prognosis of STS patients (3). The lung is the most common organ of metastasis. STS metastasis accounts for 80% of the first metastasis sites of STS, thus emphasizing the critical role of pulmonary therapy in patients with the disease (3, 4). The risk of metastasis depends largely on tumor grade; approximately 60% of patients with highly malignant tumors will develop lung metastasis (LM), the vast majority of which occur within 2 years after resection of the primary tumor (5). The highest-risk tumors are those >5 cm in size and of intermediate or high grade (6). The median life expectancy of STS patients with LM has been reported to be 10–12 months (7, 8). Given the rapid progression of high-grade STS, timely detection of LM may improve prognosis based on currently available therapeutic interventions. Despite their diversity, sarcomas are relatively rare, accounting for approximately 1% of all cancer diagnoses (9).

Treatment strategies for sarcoma lung metastases are mainly based on limited evidence due to the lack of large randomized trials. However, surgical resection of resectable metastatic tumors remains the mainstream treatment approach (10). The gold standard for patients with localized STS is surgical-wide resection of the tumor margins (2, 11). 2,9 In contrast, STS patients with metastatic disease receive systemic chemotherapy, radiation therapy, and possible resection of the primary tumor or metastatic disease (12, 13). Doxorubicin is a first-line palliative chemotherapy and is recommended as a treatment option for most STS patients with LM (14). Pulmonary metastasectomy is also a well-established standard method for treating STS patients with LM, and some published studies have shown that the 5-year overall survival (OS) rate of patients undergoing this surgery ranges from 43% to 43%. 50.9% (15–17). Furthermore, Matsuoka et al. reported that surgical resection of primary tumors in the extremities improves survival in metastatic STS (18). Despite efforts to improve clinical outcomes in these patients, prognosis remains poor (19). The effectiveness of metastasectomy in improving patient prognosis and survival is an area of ongoing research and the focus of our current study.

In addition to primary sarcomas, the metastatic behavior of these tumors is an important area of concern. Metastatic sarcomas, particularly those that metastasize to the lungs, present significant challenges in management and treatment. The complexity of this disease, coupled with the lack of specific clinical manifestations, often leads to delays or misdiagnosis, further complicating the treatment process. Several published studies have evaluated LM-related risk factors and prognostic factors in STS patients with LM, including number of LMs, histology, stage, location of primary disease, and pulmonary metastasis resection (8, 19, 20). However, to date, there is a lack of standard predictive models predicting risk and prognosis of STS vs. LM for clinicians. Therefore, this study aimed to elucidate current treatment strategies for sarcoma lung metastases, the role of metastasectomy in patient prognosis, and the challenges of diagnosing and treating this complex disease. Through this comprehensive review of the latest research and clinical practice, we hope to contribute to the ongoing discussion on improving outcomes for patients with sarcoma lung metastases.

In addition, this study is a survey of cases from the National Taiwan University Hospital. To compare with the results of past studies, this study summarizes the median postoperative outcomes of pulmonary metastasectomy and lymph node dissection in the past ten years. The survival period is shown in Table 1.

**Table 1:** Characteristics and results of relevant studies.

References	Number of patients	Patients with lymphadenectomy (%)	Prevalence of metastatic lymph nodes (%)	Solitary pulmonary metastasis (%)	Sublobar resection (%)	Adjuvant chemotherapy (%)	Follow-up (months)	Five-year survival N0 (%)	Five-year survival Nþ (%)
(21)	165	100	22	49.1	73.3	N/S	36.0	59.0	23.0
(22)	320	100	44	49.4	N/S	N/S	33.0	N/S	N/S
(23)	139	70	12	47.7	73.9	21.6	48.0	49.0	13.0
(24)	88	57	18	81.2	70.1	61.7	37.0	72.6	30.5
(25)	265	63	20	61.0	56.9	26.9	43.1	49.0	19.0
(26)	77	88	12	N/S	78.7	N/S	N/S	56.5	22.0
(27)	33	N/S	N/S	N/S	N/S	N/S	N/S	86.5	N/S
(28)	281	N/S	N/S	N/S	N/S	N/S	N/S	88.4	N/S

## 2 Materials and methods

### 2.1 Study design

This study is a single-center, retrospective observational study evaluating survival outcomes in patients who have undergone metastasectomy. We reviewed the data of patients who underwent LM metastasectomy at the National Taiwan University Hospital (including branches) from February 2007 to November 2020. The items discussed and analyzed included data of patients with sarcoma lung metastases who underwent metastasis resection such as demographic, clinical, and postoperative pathological characteristics of patients undergoing pulmonary metastasectomy, perioperative outcomes of patients undergoing metastasectomy, and histological subtypes of patients with metastatic sarcoma. These characteristics included the patient's physiological background information, whether he or she has a smoking habit, the duration of postoperative symptom-free disease, whether all the patient's pulmonary metastatic nodules had been completely resected, the location of the primary tumor, the maximum size of metastatic nodules, the laterality of lung metastases, and the surgical method.

In the National Taiwan University Hospital (including branches), the resection criteria for LM metastases are in principle based on the National Comprehensive Cancer Network (NCCN) guidelines (13) and include the following: primary cancer control, whether the lung lesions are resectable, whether the patients can tolerate pneumonectomy, and whether the patient does not have unresectable extrapulmonary disease. When patients do not meet these criteria, physicians recommend systemic chemotherapy with LM. When a patient begins LM treatment, the patient's treatment plan is determined based on discussions between the cancer surgeon and medical oncologist. Since the data for this study come from the database of National Taiwan University Hospital and the names of the patients are not disclosed, that is, the data are non-identifiable, so cases within the statistical interval are included in the analysis of this study.

### 2.2 Patients

This study conducted a retrospective analysis of patients treated at various branches of the National Taiwan University Hospital from February 2007 to December 2020. The patients include those who were treated at the main hospital of National Taiwan University Hospital from February 2007 to December 2020; at the Yunlin branch from February 2020 to December 2020; at the Yunlin branch from January 2014 to December 2020; and at the Hsinchu Branch From November 2014 to December 2020. The Research Ethics Committee of National Taiwan University Hospital approved this retrospective study (Project Approval Number: 202103065RIND).

Eligibility for inclusion in the study required patients to meet the following criteria: (1) be 18 years or older, (2) have a pathological diagnosis of sarcoma malignancy, and (3) have undergone lung resection as a metastasis resection suitable for lung metastases. Patients were excluded if the pathology report showed no evidence of primary sarcoma with concomitant lung metastases. All surgeries were performed at the National Taiwan University Hospital and its branches, and postoperative care continued until patients were discharged.

Prior to admission for surgery, the patient underwent a comprehensive preoperative evaluation. This included blood tests, chest x-rays, and electrocardiograms to assess the patient's overall health. For patients diagnosed with primary sarcoma presenting lung tumors, we almost invariably perform a transthoracic biopsy to differentiate between metastatic sarcoma and primary lung cancer, except in cases where the tumor's proximity to major vessels significantly increases the risk associated with the biopsy. In such instances, after thorough discussions with the patient about the risks, we proceed directly to surgical resection. Every patient selected for surgery underwent comprehensive scanning using CT or PET to rule out metastasis beyond the lungs, ensuring a focused and effective surgical approach. The surgical method is determined based on the patient's physical condition and the distribution of the tumor. For patients scheduled to undergo lobectomy, additional preoperative evaluation included pulmonary function testing and echocardiography to assess whether the patient's heart and lungs were suitable for surgery (29).

**Figure 1:**
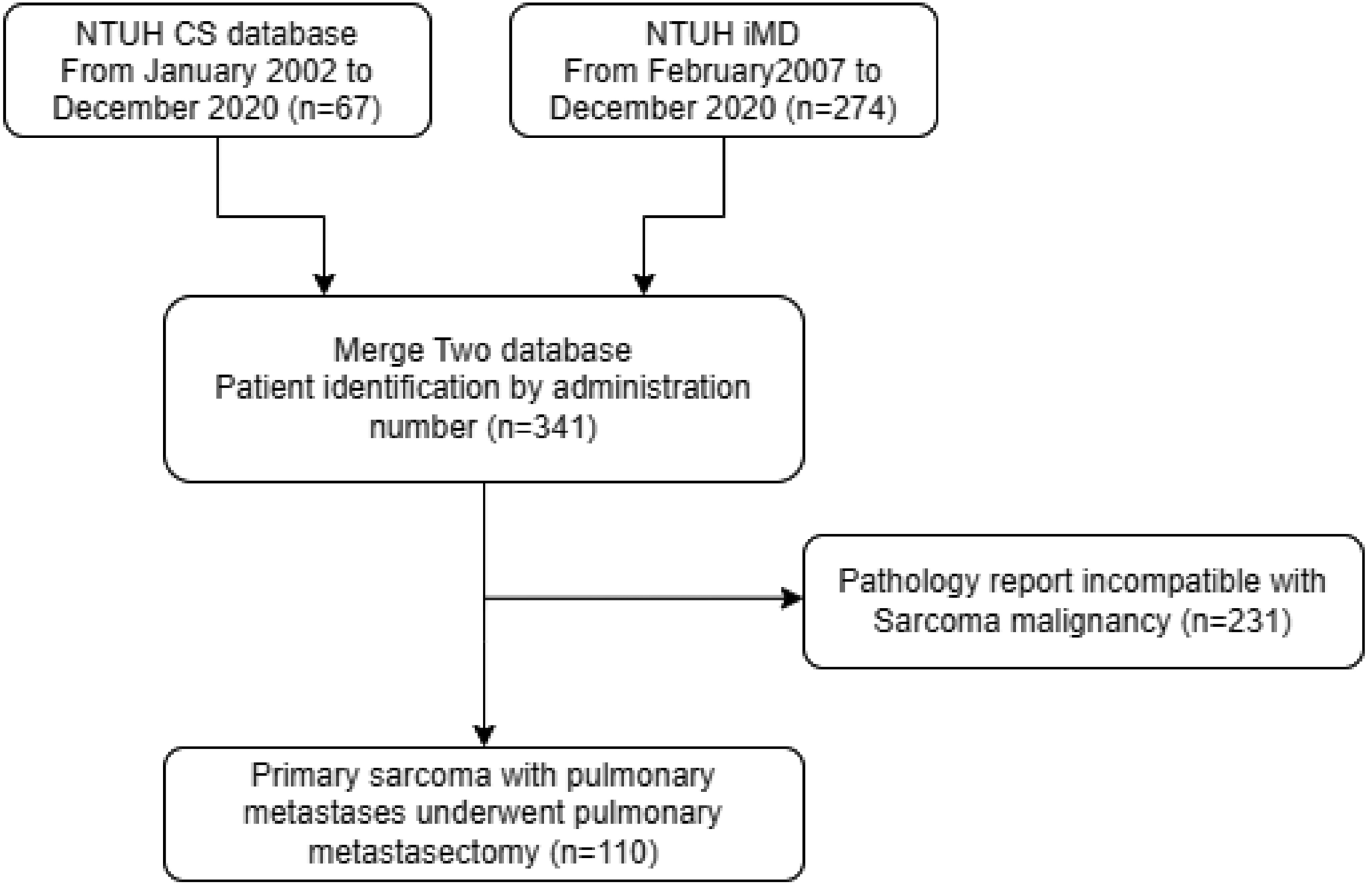
Algorithm for patient selection. NTUH, National Taiwan University Hospital; iMD, Integrative Medical Database.

The screening principles for the patients in this study are detailed in Figure 1. The samples were obtained from two pathology databases at National Taiwan University Hospital. During the 18-year period, the initial screening results showed that patients with lung metastases had been treated in our hospital. A total of 341 patients underwent resection, and samples with incomplete pathological data were further removed. Finally, a total of 110 patients were obtained as the sample number for this study.

### 2.3 Data collection

Clinicopathological information on patients, primary sarcomas, and LM was obtained from medical records. After resection of the primary tumor or LM, follow-up was performed according to NCCN guidelines. LM was diagnosed through chest computed tomography (CT) by multiple doctors, including radiologists. The date of pulmonary recurrence was recorded as the date when LM was first detected on retrospective chest CT.

When patients developed LM at the time of primary tumor resection, we defined LM as synchronous LM and recorded the date of lung recurrence as the date of surgery. The disease-free interval (DFI) was calculated from the date of knot surgery to the date of pulmonary recurrence. If the patient had synchronous LM, the DFI was recorded as 0. If the patient had multiple LM, the diameter of the largest tumor was used as the metastatic tumor size. For patients receiving chemotherapy, tumor markers were measured monthly and CT scans were performed every 3–4 months to assess disease progression during treatment. The response of LM to chemotherapy was evaluated according to the Response Evaluation Criteria in Solid Tumors version 1.1 (30). Adverse events were classified according to the National Cancer Institute Common Terminology Criteria for Adverse Events version 5.0 (31).

### 2.4 Outcomes

The primary outcomes were 5-year overall survival (OS), cancer-specific survival (CSS), and disease-free survival (DFS) in patients treated with pulmonary metastasectomy. The secondary outcome was to identify factors that have a significant impact on OS. We discussed the impact of different surgical methods on patient prognosis. In addition, the subtype distribution of sarcoma lung metastases in Asia, Europe, and America was discussed.

### 2.5 Statistical analysis

First, descriptive statistics were used to describe the ethnic characteristics of the study patients, and numerical variables are expressed as mean ± standard deviation. Categorical variables are shown as counts (percentages). Five-year OS, CSS, DFS, and progression-free survival (PFS) were then assessed using the Kaplan-Meier method, and univariate analyses were performed using the log-rank test. The distribution of characteristics between surgical and chemotherapy patients was assessed using Fisher's exact test for categorical variables and Student's *t*-test for numerical variables. All data were analyzed using Cox regression models for multivariate analysis. All statistical analyses were performed using IBM SPSS Statistics 26.0. All *p* values are two-sided, and differences were considered statistically significant at *p* < 0.05.

## 3 Results

### 3.1 Patient characteristics

This study conducted an in-depth analysis of the demographic and clinical characteristics of 110 patients diagnosed with pulmonary metastatic sarcoma. The clinicopathological data of the 110 patients are shown in Tables 2, 3. These patients have been treated in a certain institution and its affiliated branches since January 2002. As of December 2020. The average age of these patients was 48.5 years, with an almost equal distribution of men and women. The majority (70.9%) of these patients did well with an Eastern Cooperative Oncology Group (ECOG) score of 0. 18.3% of patients were smokers and 81.7% were non-smokers. In addition, 33.6% of patients had underlying comorbidities, including diabetes (11.6%), hypertension (23.9%), and other types of cancer (6.6%).

**Table 2:** Demographic and clinical features of sarcoma patients with lung metastasis patients who underwent metastasectomy.

Characteristics		All patients (%)
Age (± SD years)	Median [range]	48.5 (17.3)
	Male	54 (49.1%)
	Female	56 (50.9%)
ECOG	0	78 (70.9%)
	1	26 (23.6%)
	2	5 (4.5%)
	3	1 (0.9%)
Smoking status	Smoker	20 (18.3%)
	Non-smoker	89 (81.7%)
Comorbidities^a^		37 (33.6%)
	DM	14 (11.6%)
	HTN	29 (23.9%)
	Other cancer^b^	8 (6.6%)

^a^ 9 patients had both DM and HTN Comorbidities.

^b^ 1 patient had Lung adenocarcinoma; 1 patient had AML; 1 patient had Nasopharyngeal carcinoma; 1 patient had meningioma; 1 patient had renal cell carcinoma; 1 patient had Colon adenocarcinoma; 1 patient had breast cancer; 1 patient had urothelial carcinoma.

**Table 3:** Clinical features at the time of lung metastasectomy and pathological features of sarcoma patients with lung metastasis patients who underwent metastasectomy.

Characteristics		All patients (%)
Disease-free interval (DFI) after metastasectomy, median (months)	Mean [range]	6.6 [0–131.6]
Overall survival, median (months)		21.3
Sarcoma local recurrence at the time of lung metastasectomy		41 (37.3%)
Complete resection of all metastatic nodules		61 (55.5%)
Number of metastatic nodules	1	39 (35.5%)
	≥2	71 (64.5%)
Maximum size of the metastatic nodules (cm)		3.17 ± 3.15
Laterality	Unilateral metastasis	58 (52.7%)
	Bilateral metastasis	52 (47.3%)
Resection margin	Negative	90 (81.8%)
	Positive	6 (5.5%)
	Loss data	14 (12.7%)

During the resection of pulmonary metastases, some important pathological and clinical features were revealed. A significant 64.5% of patients had multiple (more than two) lung metastases, emphasizing the aggressive nature of the disease. Bilateral metastases occur in approximately half of patients, indicating the systemic nature of sarcoma metastasis. Consistent with contemporary trends in thoracic surgery, the majority of patients (84.6%) underwent sublobar resection and the majority (83.6%) underwent VATS. One-third of VATS procedures are single-port VATS procedures. According to the postoperative pathology report of our hospital, 81.8% of the surgeries achieved R0 resection, with ideal oncological results.

In addition, the surgical methods performed on the patients are summarized in Table 4. Among them, 23.2% of the patients underwent sequential resection of bilateral pulmonary metastases. The average postoperative hospital stay was 8.5 days, and the average ICU stay was 1.1 days. The rate of serious complications among the 110 patients after surgery was low at 4.5%, and there was no death record among all patients within 30 days after surgery.

**Table 4:** Perioperative outcomes of sarcoma patients with lung metastasis patients who underwent metastasectomy.

Treatment methods		N(%)/Mean ± SD (range)
Surgical method	Sublobar resection	93 (84.6%)
	Lobectomy	16 (14.5%)
	Bilobectomy, Pneumonectomy	1 (0.9%)
Approach method^a^	Thoracotomy	17 (15.4%)
	VATS	92 (83.6%)
	Uniportal VATS	38 (34.5%)
Sequential metastasectomy for bilateral metastasis		26 (23.2%)
Non-intubated anesthesia		10 (9.0%)
Length of hospital stay (day)		8.5 ± 6.7 (2–41)
Postoperative ICU stay (day)		1.1 ± 2.6 (0–20)
Severe morbidities		5 (4.5%)
	Prolonged air leak	4 (3.6%)
	Shock	1 (0.9%)
30-day mortality		0 (0.0%)

^a^ 1 patient underwent sternotomy method.

### 3.2 Factors correlated with overall survival

**Figure 2:**
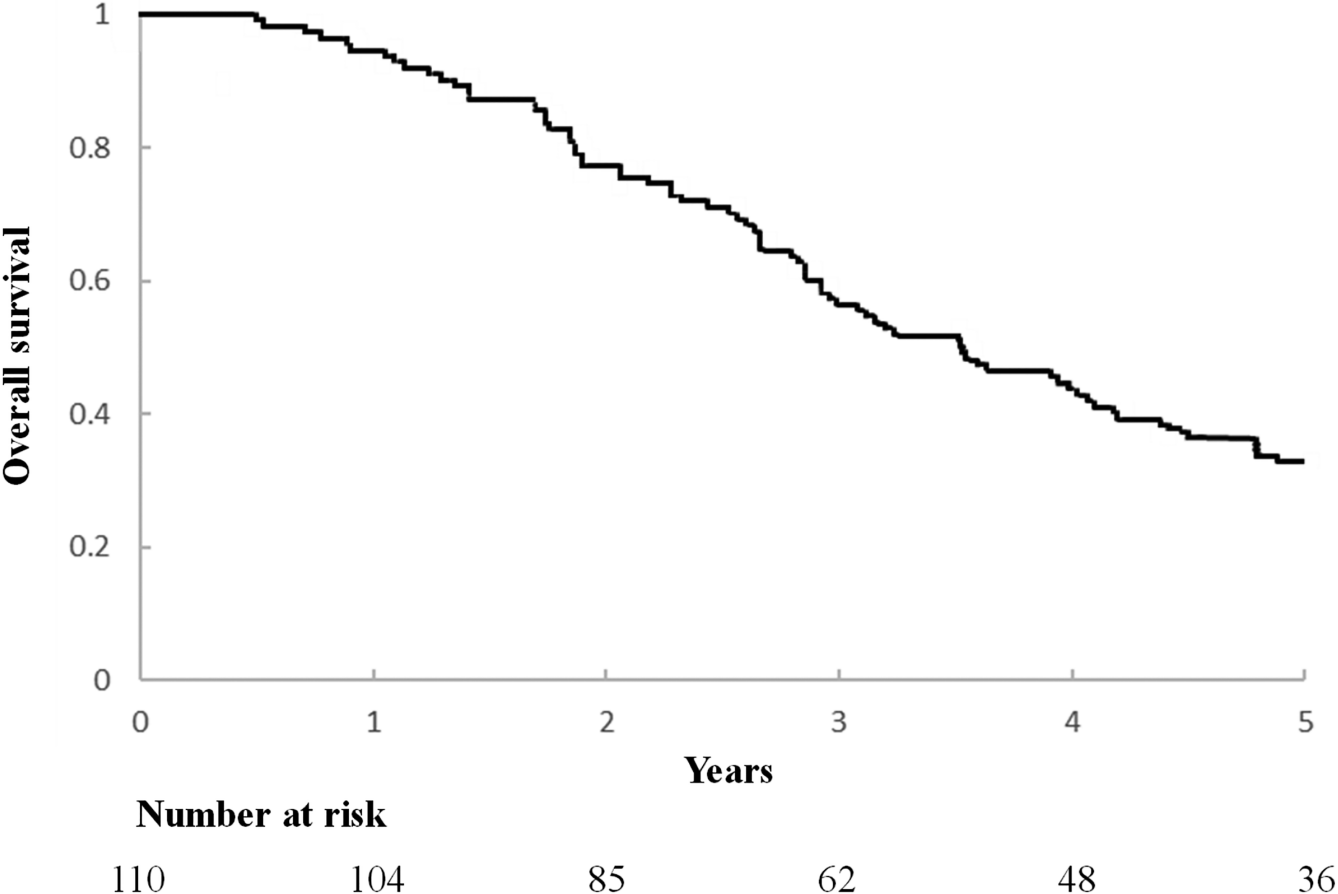
Kaplan-Meier curve of overall survival after lung metastasis surgery.

An important component of this study is the assessment of overall survival and potential influencing factors. The 5-year survival rate of surgical patients was observed to be 62.9% (Figure 2), which is a significant improvement compared with the results of previous studies. Additionally, this study used a comprehensive Kaplan-Meier analysis to identify several factors associated with improved overall survival, including a disease-free interval of more than 12 months after lung surgery, the absence of local sarcoma recurrence at the time of lung metastasectomy, and the presence of Single pulmonary metastasis tuberculosis. The results of univariate analysis of long-term prognosis are shown in Table 5. Among surgical patients, including factors such as DFI >12 months (*P* = 0.01), whether the sarcoma recurred during lung metastasis resection (*P* = 0.009), and whether the number of metastatic nodules exceeded 2 (*P* = 0.037), in this study Both are considered to have a significant impact on patient prognosis and OS. However, we further conducted multivariate analysis on the medical data of 110 patients. The results of the analysis are detailed in Table 6. We confirmed that only a disease-free interval of more than 12 months after lung surgery and the absence of local sarcoma recurrence during lung metastasis resection were significantly associated with overall survival (*P* < 0.05), this result is the same as the result of univariate analysis.

**Table 5:** Univariate analysis of correlations between clinicopathological features and OS for sarcoma patients with lung metastasis patients who underwent metastasectomy.

Variables	Patient number	Hazard ratio	95% CI	*p* value
Age, years				
<65	90	1		
≥65	20	0.519	0.218–1.236	0.139
Sex				
Male	54	1		
Female	56	0.996	0.545–1.820	0.989
ECOG				
0	78	1		
>=1	32	0.509	0.254–1.018	0.056
Smoking status				
Non-smoker	90	1		
Smoker	20	1.427	0.677–3.007	0.350
Comorbidities^a^				
Absent	74	1		
Present	36	0.826	0.431–1.584	0.566
Disease-free interval after lung surgery				
<12 months	84	1		
≥12 months	26	0.241	0.101–0.578	0.01
Sarcoma local recurrence at the time of lung metastasectomy				
Absent	69	1		
Present	41	2.226	1.218–4.068	0.009
Complete resection of all metastatic lung nodules				
No	49	1		
Yes	61	0.844	0.461–1.544	0.581
Primary tumor site				
Trunk	61	1		
Extremities	49	0.836	0.459–1.521	0.557
Number of metastatic nodules				
1	39	1		
≥2	71	1.959	1.041–3.687	0.037
Maximum size of the metastatic nodules				
<3cm	69	1		
≥3cm	39	1.047	0.567–1.935	0.884
Laterality of lung metastasis				
Unilateral	58	1		
Bilateral	52	1.648	0.905–3.000	0.103
Surgical method				
Sublobar resection	94	1		
Lobectomy/bilobectomy	16	1.057	0.488–2.289	0.887
Approach method				
Thoracotomy	17	1		
VATS	92	0.666	0.325–1.366	0.267
Margin^b^				
Free	90	1		
Involved	6	1.280	0.302–5.421	0.737

CI, confidence interval; ECOG, Eastern Cooperative Oncology Group performance status; LN, lymph nodes; OS, overall survival; VATS, video-assisted thoracoscopic surgery.

^a^ 16 patients had hypertension; 4 patients had diabetes; 1 patient had lung adenocarcinoma; 1 patient had AML; 1 patient had nasopharyngeal carcinoma; 1 patient had meningioma; 1 patient had renal cell carcinoma; 1 patient had colon adenocarcinoma; 1 patient had breast cancer; 1 patient had urothelial carcinoma; 9 patients had both DM and HTN comorbidities.

^b^ 14 patients lack the data of pathological margin.

**Table 6:** Multivariate analysis of correlations between clinicopathological features and OS for sarcoma patients with lung metastasis patients who underwent metastasectomy.

Variables	Patient number	Hazard ratio	95% CI	*p* value
Disease-free interval after lung surgery				
<12 months	84	1		
≥12 months	26	0.267	0.110–0.652	0.004
Sarcoma local recurrence at the time of lung metastasectomy				
Absent	69	1		
Present	41	1.901	1.032–3.502	0.039
Number of metastatic nodules				
1	39	1		
≥2	71	1.841	0.967–3.505	0.063

The histological subtypes of metastatic sarcomas in this study are shown in Table 7. Consistent with the results of past research (10), the most common histological subtype was leiomyosarcoma (23.6%), followed by osteosarcoma (20.0%). These findings provide important insights into treatment strategies for patients with lung metastatic sarcoma and lay the foundation for further research.

**Table 7:** Histological subtype of metastatic sarcoma patients.

Histology	No. of patient (%)
Leiomyosarcoma	26 (23.6)
Osteosarcoma	22 (20.0)
Liposarcoma	13 (11.8)
Pleomorphic sarcoma	7 (6.3)
Angiosarcoma	4 (3.6)
Synovial sarcoma	4 (3.6)
Chondrosarcoma	3 (2.7)
Spindle cell sarcoma	3 (2.7)
Rhabdomyosarcoma	3 (2.7)
Epithelioid sarcoma	3 (2.7)
Fibroblastic sarcoma	2 (1.8)
Malignant solitary fibrous tumor	2 (1.8)
Myxofibrosarcoma	2 (1.8)
Alveolar soft part sarcoma	2 (1.8)
Malignant peripheral nerve sheath tumor	2 (1.8)
Kaposi's sarcoma	1 (0.9)
Other	11 (10.0)

Among the 110 patients, 17 had bilateral metastases and underwent secondary surgeries within three months. In addition, another important finding was that a total of 92 of the 110 patients in this study underwent VATS minimally invasive surgery. After treatment, compared with the other 18 patients (one patient underwent sternotomy) who used traditional thoracotomy surgery, they had a higher survival rate within one year, and their survival time was also relatively longer.

Taken together, the comprehensive analysis of this study provides a comprehensive understanding of the patient experience, from demographic and clinical characteristics to overall survival. The presented tables also provide an organized and comprehensive view of the analyzed data, helping to understand correlations and findings more easily.

## 4 Discussion

This study provides a comprehensive analysis of clinical characteristics and outcomes in patients diagnosed with pulmonary metastatic sarcoma who underwent metastasectomy. The results of this study indicate a higher 5-year survival rate of 62.9% compared to prior reports, which ranged from 15% to 50.9% (10, 32–34). This difference in survival rates may be due to several factors, including advances in surgical techniques, perioperative care, and patient selection. It is worth noting that the results are based on patients treated in a single institute, which might have implications for the generalizability of the findings.

Our study is unique in its focus on an Asian population, a demographic that is often underrepresented in sarcoma research. The distribution of sarcoma subtypes varies between different ethnic and geographical groups, which can influence survival outcomes. Our analysis of Asian patients demonstrated that a disease-free interval over 12 months and the absence of local sarcoma recurrence at the time of lung metastasectomy significantly improved overall survival outcomes. Patients with a single lung metastatic nodule tended to have better overall survival outcomes compared to those with more than one metastatic nodule. This finding is noteworthy, although it did not reach statistical significance in the multivariate analysis in our study.

The selection of VATS or thoracotomy in our study correlates with the tumor's location, size, and the era of surgical advancements. We focused on smaller, peripherally located tumors for VATS, which coincides with the period when thoracic surgery was transitioning to the minimally invasive technique of VATS. This change overlaps with the time our study was conducted, reflecting the growing proficiency and preference for VATS among surgeons as their familiarity with the technique increased. In our practice, all patients undergo preoperative pulmonary function testing. The surgical indications for bilateral pulmonary metastasectomy are as follows: When the physician assesses that both sides of the pulmonary lesions can potentially be completely resected through staged surgeries, and the patient's lung function is evaluated to be sufficient to undergo surgeries on both sides. Our data indicate that the overall survival (OS) of patients who undergo bilateral pulmonary resections does not show significant differences compared to those with unilateral pulmonary metastases, except in cases of single-lung metastases, where a statistically significant difference was observed. However, since most of the procedures were performed using VATS, there was no notable difference in postoperative recovery time (hospital stay) or the patients’ ability to return to daily activities. All patients were able to walk and return to outpatient follow-ups after discharge.

In line with previous research, the most common histological subtype in our study was leiomyosarcoma (23.6%) (10, 35, 36). However, the frequency of this subtype was lower than that reported by some previous studies, possibly owing to the different study populations and geographical differences in sarcoma subtypes (37).

An important finding from this study was that a disease-free interval of >12 months post-lung surgery and the absence of local sarcoma recurrence at the time of lung metastasectomy were significantly associated with overall survival. These results are consistent with previous research, underscoring the importance of early detection and resection of lung metastases in improving the prognosis of sarcoma patients (35).

Our study further showed that 81.8% of surgeries achieved R0 resection, an ideal oncological outcome. This high rate of R0 resection may partly explain the higher survival rate observed in our study compared to previous reports, as complete resection of metastatic lesions has been associated with long-term survival (35). The majority of our surgeries were performed using minimal invasive surgery (VATS), which resulted in a shorter hospital stay and better postoperative recovery for most patients (38).

With the advancements in immunotherapy, particularly immune checkpoint inhibitors, there is emerging evidence supporting its use in specific sarcoma subtypes (39). While our study primarily focuses on surgical treatment, we acknowledge the potential for combining surgery with immunotherapy to enhance outcomes for sarcoma patients.

However, our study has several limitations. It is a single-arm, single-center retrospective study, which may limit the generalizability of our findings. The retrospective nature of the study may have led to some data loss, and the lack of randomization could have introduced selection bias. Furthermore, the study was conducted in a single center, which may have limited the diversity of the patient population and the applicability of the results to other settings.

## 5 Conclusions

Patients who underwent VATS minimally invasive pulmonary metastasectomy showed good long-term survival rates. In addition, statistical results on 110 samples showed that several important factors including disease-free interval after lung surgery exceeding 12 months, no local sarcoma recurrence during lung metastasis resection, and two or more tuberculosis metastases to the lungs significantly affect the survival rate of patients undergoing surgery.

Furthermore, while this study contributes valuable insights into the clinical characteristics and outcomes of patients with pulmonary metastatic sarcoma undergoing metastasectomy, further multi-center studies with larger sample sizes are needed to validate these findings. Moreover, future research should investigate the potential role of adjuvant therapies and personalized medicine approaches in improving the outcomes of these patients.

## Data Availability

The datasets presented in this study can be found in online repositories. The names of the repository/repositories and accession number(s) can be found in the article/Supplementary Material.
